# Analysis of overlapping genetic association in type 1 and type 2 diabetes

**DOI:** 10.1007/s00125-021-05428-0

**Published:** 2021-04-08

**Authors:** Jamie R. J. Inshaw, Carlo Sidore, Francesco Cucca, M. Irina Stefana, Daniel J. M. Crouch, Mark I. McCarthy, Anubha Mahajan, John A. Todd

**Affiliations:** 1 JDRF/Wellcome Diabetes and Inflammation Laboratory, Wellcome Centre for Human Genetics, Nuffield Department of Medicine, University of Oxford, Oxford, UK; 2 Institute for Research in Genetics and Biomedicine (IRGB), Cagliari, Sardinia Italy; 3 Wellcome Centre for Human Genetics, Nuffield Department of Medicine, University of Oxford, Oxford, UK; 4 Present Address: Genentech, South San Francisco, CA USA

**Keywords:** Analyses, Co-localisation, Genetics, Genome-wide association study, Insulin, Statistics, Systematic, Type 1 diabetes, Type 2 diabetes

## Abstract

**Aims/hypothesis:**

Given the potential shared aetiology between type 1 and type 2 diabetes, we aimed to identify any genetic regions associated with both diseases. For associations where there is a shared signal and the allele that increases risk to one disease also increases risk to the other, inference about shared aetiology could be made, with the potential to develop therapeutic strategies to treat or prevent both diseases simultaneously. Alternatively, if a genetic signal co-localises with divergent effect directions, it could provide valuable biological insight into how the association affects the two diseases differently.

**Methods:**

Using publicly available type 2 diabetes summary statistics from a genome-wide association study (GWAS) meta-analysis of European ancestry individuals (74,124 cases and 824,006 controls) and type 1 diabetes GWAS summary statistics from a meta-analysis of studies on individuals from the UK and Sardinia (7467 cases and 10,218 controls), we identified all regions of 0.5 Mb that contained variants associated with both diseases (false discovery rate <0.01). In each region, we performed forward stepwise logistic regression to identify independent association signals, then examined co-localisation of each type 1 diabetes signal with each type 2 diabetes signal using *coloc*. Any association with a co-localisation posterior probability of ≥0.9 was considered a genuine shared association with both diseases.

**Results:**

Of the 81 association signals from 42 genetic regions that showed association with both type 1 and type 2 diabetes, four association signals co-localised between both diseases (posterior probability ≥0.9): (1) chromosome 16q23.1, near *CTRB1*/*BCAR1*, which has been previously identified; (2) chromosome 11p15.5, near the *INS* gene; (3) chromosome 4p16.3, near *TMEM129* and (4) chromosome 1p31.3, near *PGM1*. In each of these regions, the effect of genetic variants on type 1 diabetes was in the opposite direction to the effect on type 2 diabetes. Use of additional datasets also supported the previously identified co-localisation on chromosome 9p24.2, near the *GLIS3* gene, in this case with a concordant direction of effect.

**Conclusions/interpretation:**

Four of five association signals that co-localise between type 1 diabetes and type 2 diabetes are in opposite directions, suggesting a complex genetic relationship between the two diseases.

**Graphical abstract:**

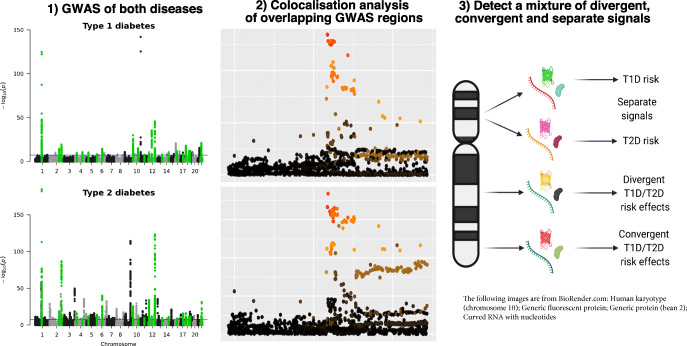

**Supplementary Information:**

The online version contains peer-reviewed but unedited supplementary material available at 10.1007/s00125-021-05428-0.

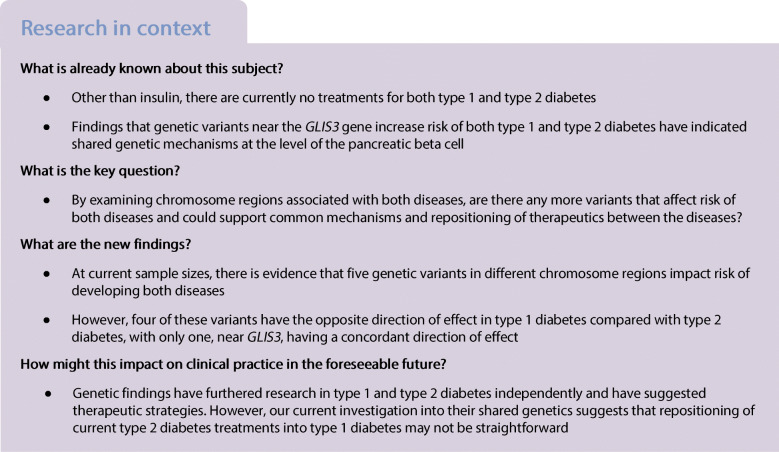



## Introduction

There is a genetic component to both type 1 and type 2 diabetes, with approximately 60 chromosome regions associated with type 1 diabetes [1] and over 200 associated with type 2 diabetes [2] at genome-wide significance. Examination of regions associated with both diseases could uncover signals that simultaneously alter disease risk for both diseases, termed co-localisation. Uncovering co-localising signals could provide biological insights into shared disease mechanisms, and potentially reveal therapeutic targets effective for both diseases. A recent analysis suggested that the same genetic variant alters risk of both type 1 and type 2 diabetes in five regions, near *CENPW*, *CTRB1*/*BCAR1*, *GLIS3*, *BCL11A* and *THADA* [3].

Here, we identified all regions across the genome that showed evidence of association with both type 1 and type 2 diseases at a false discovery rate (FDR) <0.01 and assessed co-localisation between the two diseases in each of these regions. Furthermore, to account for the possibility of multiple causal variants within an associated region, we extended the analysis to investigate conditionally independent associations within each region, to assess whether any of the associations with one disease co-localised with any associations in the other.

## Methods

Type 1 diabetes meta-analysis summary statistics were generated using genome-wide association study (GWAS) data from 3983 cases and 3994 controls from the UK (genotyped using the Illumina Infinium 550K platform), 1926 cases and 3342 controls from the UK (genotyped using the Affymetrix GeneChip 500K platform) and 1558 cases and 2882 controls from Sardinia (genotyped using the Affymetrix 6.0 and Illumina Omni Express platforms), totalling 7467 cases and 10,218 controls (Electronic supplementary material [ESM] Table 1). Genotypes were imputed using the Haplotype Reference Consortium reference panel for the UK collections [4], and a custom Sardinian reference panel of 3514 Sardinians for the Sardinian collection (ESM, Imputation).

Summary statistics for type 2 diabetes were from 74,124 cases and 824,006 controls of European ancestry, imputed using the Haplotype Reference Consortium reference panel [2].

Regions associated with both diseases were identified by selecting all variants with type 1 diabetes and a type 2 diabetes association with an FDR <0.01 (ESM Methods, Type 1 diabetes GWAS). In each such region, windows of approximately 0.5 Mb were taken to examine co-localisation (ESM Methods, Regions associated with both diseases). Within these regions, forward stepwise logistic regressions were carried out for both diseases, and conditional summary statistics were obtained so each conditionally independent signal from both diseases could be tested against each other for co-localisation (ESM Methods, Conditional analyses).

Co-localisation of signals was assessed using *coloc* [5], a Bayesian method that enumerates the posterior probability that the association signals in a region are shared between traits. The prior probability of association with either disease was taken to be 1×10^-4^ and the prior probability that the association signal is shared across traits was taken to be 5×10^-6^, as recommended [6]. The threshold to consider signals as co-localising was conservatively chosen at a posterior probability ≥0.9. Co-localisation was also examined using an alternative approach, as a secondary analysis, eCAVIAR [7] (ESM Methods, eCAVIAR).

Code used to carry out this analysis is available at https://github.com/jinshaw16/t1d-t2d-colocalisation.

## Results

Including conditionally independent association signals, 81 co-localisation analyses were carried out across 42 chromosomal regions that showed association with both diseases (ESM Table 2).

Four signals showed evidence of co-localisation using *coloc*, and these were also the regions with the highest *eCAVIAR* regional co-localisation posterior probabilities (ESM Table 3). The first was on chromosome 16q23.1, near *CTRB1* and *BCAR1*, with a posterior probability of co-localisation (H4PP hereafter) of 0.98 (ESM Fig. 1). The minor A allele at the type 2 diabetes index variant, rs72802342 (C>A), is protective for type 2 diabetes (OR 0.87, *p*=4.00×10^-32^) and susceptible for type 1 diabetes (OR 1.33, *p*=5.81×10^-10^).

The second was on chromosome 11p15.5, near *INS*, where the primary type 2 diabetes association co-localised with the secondary type 1 diabetes association (H4PP=0.95, ESM Fig. 2). The direction of effect was opposite, with the minor A allele at the type 2 diabetes index variant, rs4929965 (G>A), associated with susceptibility to type 2 diabetes (OR 1.07, *p*=4.80×10^-25^) and protection from type 1 diabetes (OR 0.87, *p*=1.89×10^-5^).

**Fig. 1:**
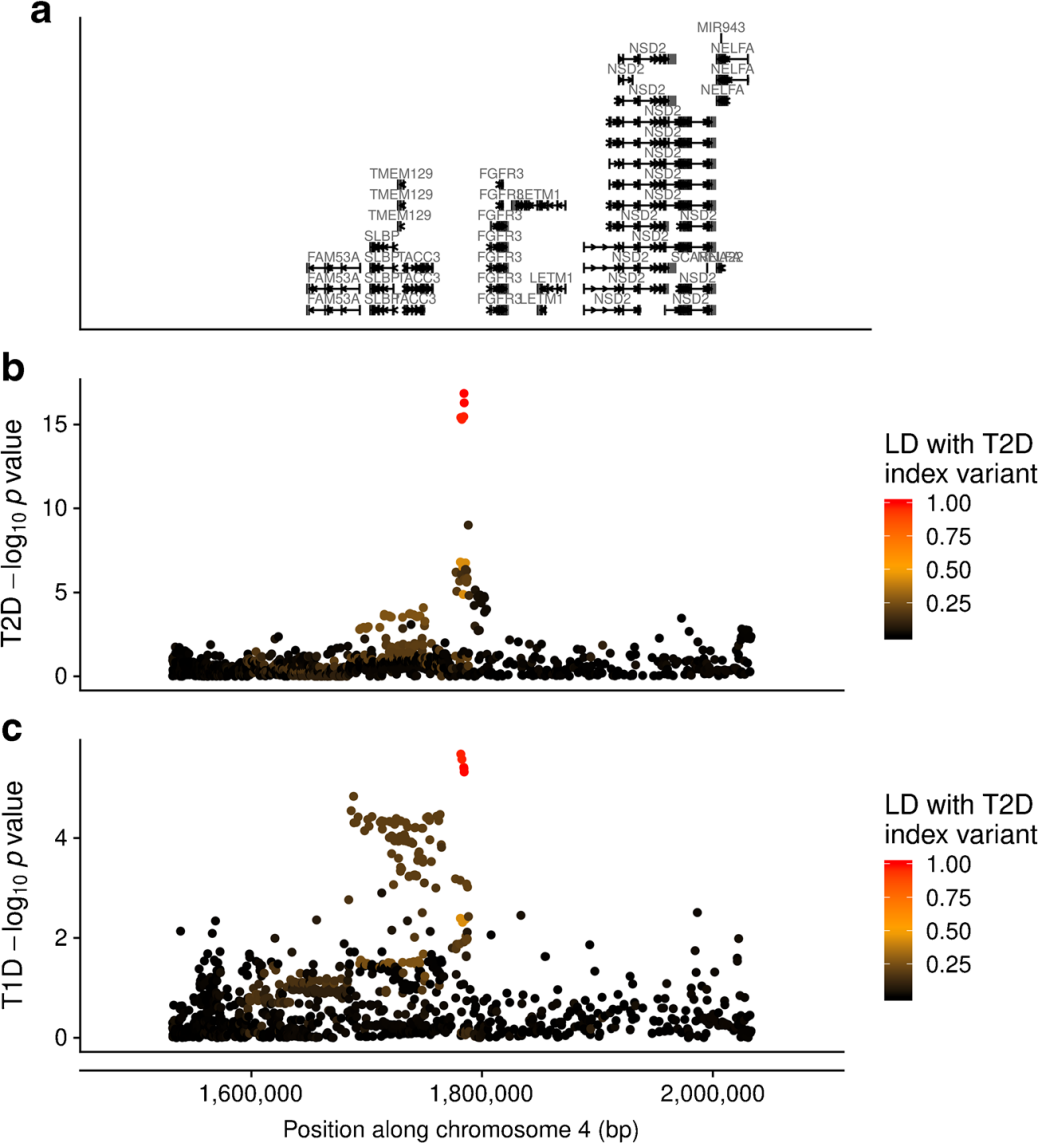
Manhattan plots showing (**a**) gene locations and –log_10_ *p* value of association for each variant by position along chromosome 4 (genome build 37) in the *TMEM129* region for (**b**) type 2 diabetes (T2D) and (**c**) type 1 diabetes (T1D), coloured by *r*^*2*^ to the type 2 diabetes index variant, rs56337234

Third, a region on chromosome 4p16.3 co-localised (H4PP=0.97) (Fig. 1), near *TMEM129*. The minor T allele at the type 2 diabetes index variant, rs56337234 (C>T), was associated with decreased risk of type 2 diabetes (OR 0.94, *p*=1.4×10^-17^) and increased risk of type 1 diabetes (OR 1.12, *p*=4.07×10^-6^).

Finally, a region on chromosome 1p31.3, near *PGM1*, co-localised (H4PP=0.91, ESM Fig. 3), with the minor T allele at the type 2 diabetes index variant rs2269247 (C>T) decreasing risk of type 2 diabetes (OR 0.96, *p*=4.6×10^-7^) and increasing risk of type 1 diabetes (OR 1.15, *p*=1.9×10^-6^) (Table 1).

**Table 1:** Regions with a co-localisation posterior probability of ≥0.9 between type 1 diabetes and type 2 diabetes

rsID	Proximal gene(s)	chr	pos (gr37)	REF	ALT	T2D conditional on:	T2D OR (95% CI)	T2D *p*	*r*^*2*^ to T1D index variant (T1D index variant)	T1D conditional on:	T1D OR (95% CI)	T1D *p*
rs2269247	*PGM1*	1p31.3	64107284	C	T	–	0.96 (0.94, 0.97)	4.60×10^-7^	0.86 (rs2269246)	–	1.14 (1.08, 1.22)	1.94×10^-6^
rs56337234	*TMEM129*	4p16.3	1784403	C	T	–	0.94 (0.93, 0.96)	1.40×10^-17^	0.97 (rs6829631)	–	1.12 (1.07, 1.18)	4.07×10^-6^
rs4929965	*INS*	11p15.5	2197286	G	A	rs11042596, rs555759341, rs571342427, rs10838787	1.07 (1.06, 1.09)	4.80×10^-25^	0.97 (rs7119275)	rs689	0.87 (0.81, 0.93)	1.89×10^-5^
rs72802342	*CTRB1/* *BCAR1*	16q23.1	75234872	C	A	rs3115960	0.87 (0.85, 0.89)	4.00×10^-32^	0.89 (rs55993634)	–	1.33 (1.22, 1.46)	5.81×10^-10^

Summary statistics given from the perspective of the index type 2 diabetes variant and with respect to the ALT allele

*r*^*2*^ obtained from 1000 Genomes Project European population

ALT, alternative allele; REF, reference allele; T2D, type 2 diabetes; T1D, type 1 diabetes

The OR is for the addition of an ALT allele

We did not replicate the finding that the chromosome regions near *CENPW, GLIS3, BCL11A or THADA* co-localised between type 1 and type 2 diabetes (H4PP *CENPW*=0.12, *GLIS3*=0.29, *BCL11A*=0.28, *THADA* not examined as no type 1 diabetes association existed in the region [FDR=0.07]). To investigate these discrepancies, we examined two other large type 2 diabetes meta-analyses: a trans-ethnic study including 1,407,282 individuals [8] and a study of 433,540 individuals of East Asian ancestry [9]. For the *CENPW* and *BCL11A* regions, the type 2 diabetes signal is consistent with at least one of the other GWAS studies (measured by linkage disequilibrium [LD] in Europeans to the other study index variants, ESM Table 4), and the type 1 diabetes index variant is not in strong LD (*r*^*2*^<0.41) with any of the index variants for type 2 diabetes across the three GWAS studies. However, at *GLIS3*, there appears to be a distinct signal in the European study [2] compared with the trans-ethnic and East Asian type 2 diabetes studies (*r*^*2*^=0.65), and the index variants from these two studies are in higher *r*^2^ with the type 1 diabetes signal in our analysis (*r*^*2*^=0.68), and even higher *r*^2^ with the index variant from a larger type 1 diabetes genetic analysis [1] (*r*^*2*^=0.99), indicating that the signal near *GLIS3* does co-localise between type 1 and type 2 diabetes with concordant direction of effect, as previously identified [10].

## Discussion

Using genetic association summary statistics from European populations, we identified 42 regions that showed association with both type 1 and type 2 diabetes, with 81 conditionally independent association signals across those regions. Four signals (near *CTRB1/BCAR1*, *INS*, *TMEM129* and *PGM1*) co-localised between the diseases, including a signal at the complex *INS* region for the first time, which was achieved by examining conditional summary statistics. However, in all four cases, the allele increasing risk for one disease was protective against the other. Examination of additional trans-ethnic and East Asian type 2 diabetes genetic analyses indicated that a fifth association, near *GLIS3*, is likely to co-localise between diseases, with concordant direction of effect.

Given the distinct mechanisms underlying beta cell dysfunction and cell death between the two diseases [11], it is perhaps unsurprising that no additional signals were detected with concordant direction of effect. However, the type 1 diabetes GWAS was much smaller than the type 2 diabetes analysis, and therefore had less statistical power to detect more subtle genetic effects. If a type 1 diabetes GWAS were to be performed with similar power to the type 2 diabetes GWAS, more regions might co-localise between the two diseases, but either the effects of these additional regions on type 1 diabetes would be small compared with the currently known associations or they would be rare variants with larger effect sizes.

That four of five co-localisation signals had opposite directions of effect implies a complex genetic relationship between the two diseases. While the directional discordance offers little hope for effective treatments for both diseases simultaneously at these particular targets, it can offer biological insight into the disease pathways that these regions act upon, and even if there is directional discordance, the genetics could be highlighting the same therapeutic target.

We did not replicate the findings that the associations near *BCL11A*, *CENPW* and *THADA* co-localise between the two diseases [3], despite overlapping samples and similar numbers of cases and controls in the type 1 diabetes GWAS. There are three possible reasons for this: 1) the previous study [3] examined co-localisation using weaker association signals, for example, the co-localisation near *THADA* was based on a type 1 diabetes association *p* value of 0.01; 2) we used a more stringent prior for co-localisation between the two diseases, as recently suggested [6] (5×10^-6^ vs 1×10^-5^); and 3) we used a more stringent posterior probability threshold to declare co-localisation (0.9 vs 0.5). Our increased stringency compared with the previous analysis [3], while increasing the probability that any identified shared signals will be true positives, may have decreased our sensitivity to detect all co-localisations. For example, by examining other large type 2 diabetes GWAS analyses and a larger type 1 diabetes genetic analysis, we conclude that the association near *GLIS3* likely does co-localise between the two diseases, and with concordant directions of effect.

In conclusion, with current GWAS sample sizes, just five associations appear to co-localise between type 1 diabetes and type 2 diabetes, four with opposing direction of effect. Larger sample sizes would be required to identify the depth of genetically identified therapeutic targets to treat or prevent both diseases simultaneously.

## Supplementary Information

ESM 1 (PDF 2124 kb)

ESM 2 (XLSX 43 kb)

## Data Availability

Type 1 diabetes summary statistics will be available through the GWAS catalogue (https://www.ebi.ac.uk/gwas/). Type 2 diabetes summary statistics are already publicly available.

## Abbreviations

FDR: False discovery rate
GWAS: Genome-wide association study
H4PP: Posterior probability of co-localisation
LD: Linkage disequilibrium
